# Characteristics of Canteens at Elementary Schools, Upper Secondary Schools and Workplaces that Comply with Food Service Guidelines and Have a Greater Focus on Food Waste

**DOI:** 10.3390/ijerph16071115

**Published:** 2019-03-28

**Authors:** Anne D. Lassen, Lene M. Christensen, Max P. Spooner, Ellen Trolle

**Affiliations:** 1 Division of Risk Assessment and Nutrition, National Food Institute, Technical University of Denmark, DK-2800 Lyngby, Denmark; lmch@food.dtu.dk (L.M.C.); eltr@food.dtu.dk (E.T.); 2 Department of Applied Mathematics and Computer Science, Technical University of Denmark, DK-2800 Lyngby, Denmark; mpsp@dtu.dk

**Keywords:** workplace, upper secondary schools, elementary schools, hot meals, food and nutritional environment, organic food, UN Sustainable Development Goals

## Abstract

Policy actions to improve the nutritional environment include the provision of official food service guidelines. This study aimed to examine compliance with food service guidelines for hot meals as well as self-evaluated focus on food waste reduction across settings, i.e., elementary schools, upper secondary schools and workplaces, and different canteen characteristics. The same five criteria for hot meals were applied for all settings with regard to serving of fruit and vegetables, fish, wholegrain product and high fat meat and dairy products. A self-administered questionnaire survey was conducted as a cross-sectional study among 680 Danish canteens. Canteens having a high degree of organic food procurement were more likely to comply with the five criteria for hot meals combined (OR 2.00 (Cl 1.13,3.53)). Also, the use of organic food together with having a meal policy was associated with reported focus on food waste reduction (OR 1.91 (CI 1.12,3.25) and 1.84 (Cl 1.31,2.59), respectively). Compliance with individual criteria varied across settings with elementary schools being more likely to comply with criteria on, e.g., maximum serving of non-wholegrain products, whereas workplaces were more likely to comply with criteria on, e.g., minimum fruit and vegetable content and serving of fish. In addition, specific characteristics, e.g., serving system, were found to predict compliance with some of the criteria. These findings highlight the need to address differences in canteen characteristics when planning implementation support for both guideline and food waste reduction initiatives.

## 1. Introduction

Improving diet quality while simultaneously reducing environmental impact and achieving sustainable development outcomes is a critical focus globally, both at the individual and institutional levels [1,2,3]. This includes the food service sector that plays a significant role in relation to many people’s everyday food intake. An increased availability of healthier food options has been found to positively impact dietary intake among both primary school children [4,5,6] and employees having lunch at the canteen [7,8]. Moreover, implementation and maintenance of a healthy nutritional environment has been found to be less expensive than nutrition education interventions [9].

Policy actions to improve the nutritional environment include the provision of official food service guidelines. Many countries have introduced policies and official guidelines that support the provision of healthier food and beverage options and restrict unhealthy options at canteens at schools [10,11], and to a lesser extent, also at workplaces [12,13], either voluntary or obligatory. Despite the introduction of such policies and guidelines, the reported adherence to the policies and guidelines is mixed [14,15,16], suggesting a need for research on strategies and factors underlying the success of such policies to increase adherence to food service policies and guidelines [10,17,18]. Also, this highlights the need to monitor the food environment to inform about points of progress or lack of progress towards meeting the policy goals in the different settings. In recent guidelines from Germany and England, more emphasis is put on food-based recommendations for school meals rather than focusing on the nutrient content of the meals [19,20]. For example, the UK revised standards to include five requirements in relation to the food group of meat, fish, eggs, beans and non-dairy sources of protein, primarily to ensure adequate provision of protein, iron and zinc while limiting the amount of fat, saturated fat and salt [21]. This approach might be easier for the food service operators to understand and apply in practice because it does not require access to nutrient calculation programs and up-to-date food composition data [14,22,23]. In line with this, the Danish Veterinary and Food Administration developed a new concept for food-based guidelines in 2017 directed at food service operators in elementary schools, after-school care, upper secondary schools and workplaces [24,25,26,27]. Further, in 2018, new food-based guidelines directed at daycare institutions were launched [28]. The guidelines, that are voluntary to follow, contain criteria for serving fruit and vegetables, wholegrain products and fish, and limiting high fat and high sugar products for different food categories at breakfast and lunch, e.g., hot meals, sandwiches and salads, as well as snacks, beverages and afternoon meals. Canteens that comply with all these guidelines, and in addition reduce salt and sugar content, can brand themselves with the Danish Meal Label [29]. The present study focused on hot meals served at lunch, which is a food category served by both schools and workplace canteens and therefore comparable across settings. 

In Denmark, there has not been a tradition of providing food in either elementary or upper secondary schools. Around half of Danish elementary schools is estimated to offer lunch options that the pupils can buy at lunch time [30]. A typical school hot meal might consist of chicken, a wholegrain roll, and salad. Besides hot meals, many elementary schools offer sandwiches and snack meals and to a lesser extent salad bars. Sandwiches and snack meals are likewise offered by most upper secondary schools as well as a salad bar [31]. With regard to workplaces, around a third is estimated to have an in-house canteen that offers lunch options for sale. Workplaces with more than 50 employees more often have a canteen [32]. A typical workplace hot meal might consist of roasted meat with potatoes and vegetables. In addition, most workplace canteens offer a salad bar and an open-faced sandwich buffet [31]. 

To the best of our knowledge, no studies have been conducted until now on compliance with food service guidelines across the different settings or whether compliance differs by canteen characteristics, including the use of organic food. However, some studies have examined compliance within individual settings, e.g., in other Nordic countries, the UK and Australia [14,15,16].

The market for organic food products is growing worldwide [33]. In Denmark, the use of organic food procurement in public kitchens has a long history of implementation and development [34]. In 2009, The Ministry of Food Agriculture and Fisheries introduced the Organic Cuisine Label for the marketing of organic food in kitchens. In 2012, The Danish Organic Action Plan 2020 was launched, and updated in 2015, to establish political support for organic food conversion projects targeting public kitchens [35]. Currently, nearly 2700 places in Denmark use the Organic Cuisine Label [36]. Increasing use of organic food has been suggested to result in meal compositions more in line with food-based recommendations both among professional kitchens [37,38] and among the general population [39]. Also, organic food procurement levels have been suggested to result in a greater focus on food waste reduction in Danish public kitchens [38]. Food waste may include waste generated in the kitchen when preparing and cooking the food, during serving and from customer plate leftovers. In a Danish study, two workplace canteens were found to waste about one-fifth of all food prepared in serving from a self-service buffet. This is in line with results found in the Finnish food service sector [40]. In the Danish study, the canteens almost halved their food waste during an organic food conversion project [41]. 

Environmental sustainability issues related to food waste have been an increasing concern during recent years [42,43], and the food waste issue is a key part of the UN Sustainable Development Goal number 12, “Ensure sustainable consumption and production patterns” [44]. Also, the EAT-Lancet commission concludes that “A radical transformation towards healthy diets from sustainable food systems is urgently needed”. This requires the active involvement of actors in all sectors, including the food service sector. Chefs and other culinary professionals are well positioned to minimize food waste and make healthy and sustainable foods delicious [2]. Studies on food waste in the food service sector are, however, limited. Kinasz et al. points out that more research is needed to identify the factors controlling food waste generation [45]. Martin-Rios et al. argues that reducing food waste is a key sustainability challenge for the food service industry that requires a whole new set of management practices [46].

The primary aim of the present study was to measure whether the compliance with food service criteria on hot meals at lunch varies according to setting (elementary schools, upper secondary schools and workplaces), and characteristics of the canteens. A secondary aim was to examine the self-evaluated focus on food waste reduction across settings and different canteen characteristics. The canteen characteristics include the use of organic food, having a meal policy, number of daily lunch meals served, serving system and outsourced to external contractors vs. those operated by the workplace/school.

## 2. Materials and Methods 

### 2.1. Study Design and Recruitment of Canteens

The survey was conducted as a cross-sectional study. Data were collected through three online self-administered questionnaires using the LimeSurvey open source system directed at public and private elementary schools (enrolling children aged 5–16 years), public upper secondary schools (enrolling students aged 15–19 years) and public and private workplaces. The questionnaires were alike in order to be comparable, with the exception of a few questions that only applied to, e.g., elementary schools or workplaces. An e-mail invitation with a link to the survey was sent to the canteen managers. Canteen managers not responding were e-mailed one reminder. Moreover, managers at canteens at upper secondary schools not responding also got a phone call from a member of the project staff urging them to participate due to an initial low number of canteens in this group. The questionnaire survey was carried out between September and November 2017. 

The recruitment of canteens was performed on the basis of a register of Danish canteens used by the Danish Food Inspections and held by the Danish Veterinary and Food Administration. By law, all restaurants and other enterprises selling food and beverages to the public are obliged to register at the Danish Veterinary and Food Administration. In the study, canteens preparing food in-house at elementary schools, upper secondary schools or workplaces were eligible for inclusion. The register had e-mail addresses for one third of these canteens. The remaining e-mail addresses were obtained via phone or e-mail inquiry to the workplace/schools or to the canteen operator companies. A total of 1995 canteens (367 elementary schools, 208 upper secondary schools and 1420 workplaces) across the whole country were invited to participate in the survey.

The study was performed in accordance with the ethical standards of the Helsinki Declaration of 1975, as revised in 2008. The Danish National Committee on Health Research Ethics has decided that, according to Danish Law, this kind of study does not require approval. 

### 2.2. Questionnaire Development and Content

The questionnaires were designed to evaluate the compliance of the meals in the canteens with the guidelines (see Table 1 regarding criteria for hot meals) as well as to measure the self-evaluated focus of food waste reduction. The questionnaires were developed through different phases in an iterative process, each phase and version gradually informing the next through repeated adjustments performed by the research group. Questions were initially developed based on a literature review and knowledge on content of Danish canteen meals [47,48,49,50,51]. Feedback on the initial questionnaire was acquired from nine food and nutrition professionals, i.e., representatives from the Danish Veterinary and Food Administration, food service representatives, food service nutritional consultants and nutritional researchers, including researchers in dietary assessment methods. They were asked to add comments and suggestions for overall content and relevance, comprehensibility of questions and response options, clarity of wording, missing items or any additional comments. To evaluate content validity and acceptability, the questionnaires were further tested among 13 canteens chosen to represent canteens with different characteristics and different settings (five elementary schools, three upper secondary schools and five workplaces). “Think-aloud” interviewing was conducted as they filled in the questionnaire. This is a cognitive interviewing technique wherein survey respondents are asked to actively verbalize their thoughts as they attempt to answer the evaluated survey questions [52]. In a few cases, “retrospective probing” was used due to busy time schedules among canteen managers. This is a verbal probing technique wherein the interviewer administers the probe questions after the respondent has completed the entire survey [52]. In both cases the interviewer visited the food service unit beforehand at lunch-time to be able to compare actual servings of meals and their ingredients with the respondents’ answers to the questionnaire. The actual servings were recorded by observing the food production, i.e., the ingredients and amounts used were registered in templates. Recipes and nutrition labelling of the food products used were also collected. The actual servings registered by the interviewer were compared with the answers of the questionnaire made by the canteen manager. Comparison were conducted in Excel and evaluated case by case. The feedback on the questionnaire was reviewed by one member of the project staff and checked by another. Some minor differences in the registration made by the interviewer and the canteen managers were found, however the measure of compliance to the criteria of hot meals was found to be practically similar. Finally, revised questionnaires were tested among a sample of 40 canteens using the same procedure and method as used in the final survey to test how long it took to fill out the questionnaire and to make a final evaluation of the questions. On the basis of this, some of the questions not necessary for evaluation of compliance to the guideline criteria were made non-compulsory. The respondents then had the opportunity to skip these questions and thereby shorten the response time, if this was perceived as a problem. In total, the questionnaires contained 88 (elementary schools), 89 (secondary schools) and 87 questions (workplaces) of which about 80% were compulsory. The number of questions that the individual school or workplace needed to answer depended on the number of food categories they offered. The three questionnaires including the questions on hot meals and canteen characteristics are shown in Supplementary Materials S1–S3. The response time was 25 min on average in the final survey.

**Figure 1:**
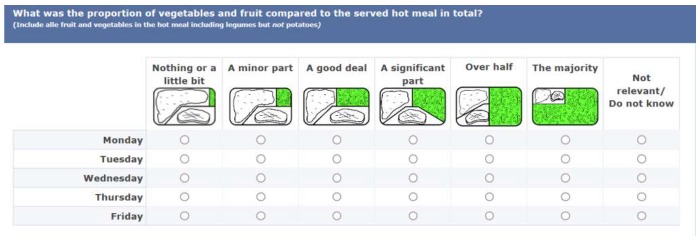
Question on the relative content of fruit and vegetables in the hot meals (Monday to Friday).

Information regarding the compliance with hot meals guidelines was obtained through four questions on hot meal contents during the last week. The criteria regarding fish and meat products were evaluated with the question: “What was the main source of protein in the hot meals (for each day last week)?” with the following answering categories: “Poultry (e.g., chicken, turkey)”, “Red meat with more than 10% fat (e.g., pork loin with rind, lamb leg, beef chuck)”, “Red meat less than 10% fat (e.g., tenderloin trimmed, minced meat lean)”, “Fish and shellfish”, “Eggs”, “Legumes (e.g., beans, lentils)” or “Other” (Monday to Friday). The criteria regarding wholegrain was evaluated with the question: “What was the main source of starch in the dish (for each day last week)?” with the following answering categories: “Potatoes”, “White rice, couscous etc.”, “Brown rice, kernels etc.”, “Pasta etc. without wholegrain”, “Wholegrain pasta etc.”, “White bread etc.”, “Wholegrain bread”, “Other” (Monday to Friday). To evaluate the criteria regarding fruit and vegetable content, the following question was asked: “What was the proportion of fruit and vegetables compared with the served hot meal in total (for each day last week)?” A scale of figures showing six amounts of fruit and vegetables were developed illustrating “Nothing or a little bit” (less than 10%), “A minor part” (about 20%), “A good deal” (about 25%), “A significant part” (about 33%), “Over half” (about 50%), “The majority” (about 75% or more) (Figure 1). 

Finally, the questionnaires also included questions on food waste reduction: “To what extent does the canteen focus on food waste?” with responses on a 5-point scale from “A very low degree” to “A very high degree”. This question was used as an indication of the canteens’ awareness and attitudes towards food waste reduction. Respondents from canteens answering “to some degree”, “to a high degree” or “to a very high degree” had the opportunity to select from a list of pre-defined options about how they work with reducing food waste. These pre-defined options were identified and selected on the basis of literature [53,54]: “Optimize the use of raw foods”(i.e., using the entire product), “place less food on the table or buffet (and rather refill it more times)”, “using smaller serving bowls”, “adapting portion sizes”, “reuse of excess production/leftovers”, “sells leftovers to employees etc.” and “Other”. The answering options represent food waste generated during the preparation of the food, serving and from customer plate leftovers. 

Questions regarding the characteristics of the settings included number of lunch meals served on a daily basis with six answering categories from “below 25” to “more than 1000”; the use of serving system for lunch—either “buffet style/self-service”, where a variety of food choices are offered at a fixed price, “a cash á la carte system”, where the customers select and purchase the items for lunch, as individually served portions or finally “served in bowls at the table”; canteen outsourced or operated by the setting; presence of a formal written canteen meal policy (yes or no); and percentage use of organic food with ten answering options from “about 0%” to “about 100%”.

### 2.3. Outcome and Explanatory Variables

Compliance and non-compliance with each of the five hot meal criteria were defined, and in addition a combined category for compliance with all five criteria. Compliance with the fruit and vegetable criteria was defined as canteens answering “A significant part”, “More than half” or “The majority” (about 1/3 of the serving or more) for at least four out of five meals. Compliance with the fish criteria was defined as canteens serving fish or shellfish at least 1 of 5 days. Compliance with the high-fat meat criteria (as main protein component) and the wholegrain criteria was defined as canteens serving meat with more than 10% fat maximum up to one of five meals, and serving either white rice, white bread or non-wholegrain varieties of pasta maximum up to one of five meals. Compliance with the criteria on high-fat meat and dairy products (besides the main protein component) was defined as using cheese over 17% fat, dairy product over 5% fat, high-fat meat to flavor the meal or solid fats up to two of five meals. Only canteens serving hot meals at least 3 days a week were included in the analysis. If hot meals were served three or four days a week the criteria for compliance were changed proportionally. With regard to food waste reduction, participants who responded “A very high degree” were included as outcome variable. 

Explanatory variables were defined as number of lunches served on a daily basis over or below 100 servings a day, serving system being either a buffet system or other serving systems, canteen outsourced to an external contractor or operated by the setting, the presence of a written canteen meal policy or not and the use of organic food over or below 50%. 

### 2.4. Statistical Analysis

Statistical analyses were performed using the statistical software package SAS version 9.4. Descriptive statistics were used to describe the characteristics of the canteens participating in the study. Multiple logistic regression models were used to investigate factors associated with fulfilment of guidelines and food waste reduction. Six variables of interest were identified: Setting, number of daily lunch meals, outsourced to external contractors, written canteen meal policy, serving system, organic food procurement. For each criteria and for food waste reduction, an initial model was fitted consisting of the six main effects. Backward model selection was then applied to sequentially remove the least significant variable, until only significant (*p* < 0.05) variables remained in the model. Potential two-way interactions between the remaining explanatory variables were then tested using the same procedure. The goodness of fit was assessed using the Hosmer-Lemeshow goodness of fit test. The sample size was considered adequate for fitting these models, according to the widely used guideline for multiple logistic regression that there should be a minimum of 10 events per investigated covariate [55,56].

## 3. Results

A total of 680 canteens out of 1995 invited answered the questionnaire, corresponding to a total response rate of 34%. The proportion of canteens participating across different Danish Regions were comparable with those invited as shown in the Supplementary Material (Table S1). In total, 86% of the respondents reported that they were canteen managers or canteen employees. The rest were managers in general or teachers at the schools.

### 3.1. Canteen Characteristics

Table 2 shows the characteristics of the participating canteens. The proportion of canteens having a written meal policy was 45%. In total, 14% of the canteens used more than 50% of organic food procurement, with the elementary schools and the upper secondary schools having the highest and lowest proportions of canteens that used more than 50% organic food procurement, respectively (22% and 9%, respectively).

### 3.2. Compliance with the Guidelines

Compliance with guidelines for hot meals is shown in Table 3 for canteens in elementary schools, upper secondary schools and workplaces, respectively. For the canteens in elementary schools, the highest compliance was seen for the criteria on the maximum frequency of serving high-fat meat as main protein component and non-wholegrain products (88% and 80%, respectively). For the canteens in the upper secondary schools, the highest compliance was seen for the criteria on maximum frequency of using high-fat meat as main protein component (81%). With regard to canteens at workplaces, the highest compliance was seen for the criteria on minimum frequency of serving fish (82%). For all settings, the lowest compliance was seen for the criteria on minimum content of fruit and vegetables (38%, 31% and 45%, respectively, for elementary schools, upper secondary schools and workplaces). Only a minor proportion of the settings complied to all of the criteria combined (19%, 10% and 13%, for elementary schools, upper secondary schools and workplaces, respectively). 

Table 4 shows the characteristics of the canteens found to be significantly associated with compliance with each of the hot meal criteria. No interactions between the canteen characteristics were found significant. Canteens with a high degree of organic food procurement (organic food procurement >50% vs. <50%: OR 1.88 (1.14,3.11)) and canteens at workplaces were more likely to comply with the criteria on minimum fruit and vegetable content than the other canteens (workplace v. elementary school: OR 1.84 (1.16,2.90) and workplace v. upper secondary school: OR 1.98 (1.17,3.34)). Moreover, workplace canteens were found to be more likely to comply with the criteria on minimum frequency of serving fish compared with other canteens (workplace v. elementary school: OR 3.58 (2.19,5.84) and workplace v. upper secondary school: OR 5.63 (3.35,9.45)). Multiple factors were found to be associated with compliance with the criteria on maximum frequency of serving high-fat meat as main protein component including being an elementary school canteen, having a high degree of organic food procurement, serving buffet style and not having af meal policy. With regard to the criteria on the maximum frequency of use of non-wholegrain products, being an elementary school canteen, serving buffet style and not being outsourced to external contractors were associated with compliance with the criteria. Moreover, elementary school canteens were more likely to comply with the criteria on the maximum use of high-fat dairy/meat products in limited amounts compared with workplace canteens (workplace v. elementary school: OR 0.47 (0.30,0.74)). Finally, canteens having a high degree of organic food procurement (>50%) were more likely to comply with the 5 criteria for hot meals combined (OR 2.00 (1.13,3.53)).

### 3.3. Food Waste Reduction

Table 5 shows answers to the question on the canteens’ focus on reducing food waste. The vast majority of the canteens reported that they had “somewhat”, “a high degree” or “a very high degree” of focus on food waste reduction (93% on average). The proportion of elementary schools reporting that they focus on food waste reduction to “a very high degree” was 62%, while 59% of upper secondary schools and 58% of workplaces reported to focus on food waste reduction “to a very high degree”.

As seen in Table 6, canteens with a high degree of organic food procurement were found to have significantly higher odds of focusing on food waste reduction to a very high degree, than the canteens with less than 50% organic food procurement (OR 1.91 (CI 1.12,3.25)). Also, canteens with a meal policy were found to have significantly higher odds of focusing on food waste reduction to a very high degree compared with canteens without a meal policy (OR 1.84 (CI 1.31,2.59)).

**Figure 2:**
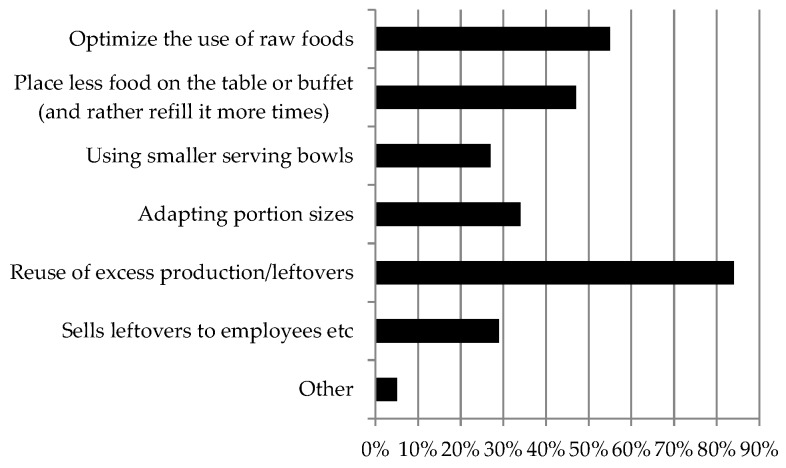
Food waste reduction priorities among canteens in elementary schools, upper secondary schools and workplaces that focus on food waste reduction “somewhat”, to “a high degree” or “a very high degree” (%) (*n* = 638).

Figure 2 shows how the canteens reported to work with reducing food waste. The single most used strategy to reduce food waste was reported to be reuse of excess production/leftovers (84%). In total, 55% answered that they optimize the use of raw foods and 47% that they place less food on the table or buffet. Adapting portion sizes and using smaller bowls were reported to be used to reduce food waste by 34% and 27%, respectively. Selling leftovers to employees was a strategy used by 29% of the canteens. Finally, 5% of the canteens had other strategies with giving away the leftovers for free, being the most often mentioned.

A very similar pattern in food waste reduction priorities was seen among upper secondary schools and workplaces. With regard to elementary schools, small differences were seen, e.g., more schools reported to reduce food waste by adapting portion sizes (40%). Canteens using organic food procurement were found to have more priorities in optimizing the use of raw foods (72%) compared with those not using organic food procurement to the same extent (52%), whereas canteens having a written canteen meal policy were found to have more priorities in adapting portion sizes (42%) and using smaller serving bowls (34%) compared with priorities among other canteens not having a written meal policy (28 and 21%, respectively).

## 4. Discussion

The present study, examining the availability of hot meals complying with the official Danish food service guidelines, shows that canteens at elementary schools, upper secondary schools and workplaces have room for improvement in order to promote healthier food choices, since only 14% of the canteens fulfilled all five criteria for hot meals over a week. Higher compliance was seen with regard to the five individual hot meal criteria from 41% (minimum fruit and vegetable content) to 75% (maximum frequency of serving high-fat meat as main protein component). 

Looking overall at the characteristics of the canteens, especially the type of setting (elementary school, upper secondary school or workplace) and the use of organic food (over or below 50%) were associated with compliance with the criteria. The type of setting was associated with compliance with all five criteria individually, and the use of organic food was associated with compliance with two of the criteria (i.e., criteria on minimum fruit and vegetable content and maximum frequency of serving high-fat meat as main protein component) as well as the five criteria combined. Besides, serving buffet style and the presence of a written meal policy, as well as serving buffet style and being outsourced to external contractors were found to be associated with compliance with two of the criteria (frequency of serving high-fat meat as main protein component, and maximum frequency of serving non-wholegrain products, respectively). Moreover, a high use of organic food and the presence of a written meal policy were found to be associated with reporting a very high degree of focus on food waste reduction.

Compliance of the individual criteria for hot meals was highly variable across the different settings. Some of the criteria for hot meals were more likely to be fulfilled by elementary schools compared with both workplaces and upper secondary schools, e.g., maximum frequency of serving high-fat meat as main protein component (88%), using high-fat dairy/meat products in the meals (62%) and frequency of serving non-wholegrain products (80%), whereas workplaces were found to be more likely to comply with the criteria of minimum serving of fish in accordance with the guidelines (82%) compared with both elementary school and upper secondary school canteens. Also workplaces were more likely to comply with the criteria on minimum fruit and vegetables content of the meals (45%) compared with both canteens at elementary schools and upper secondary schools.

Other studies in the school setting have reported mixed results with regard to compliance with food service policies and guidelines. Juniusdottir et al. found that the guidelines and recommendations on the availability of different foods were generally quite well followed, but only 61% of the meals satisfied the recommendation of minimum energy content among the 24 participating elementary schools from three Nordic countries [14]. Lower levels of compliance were found in a study among 263 primary and secondary Australian school menus, where the proportion of schools compliant with healthy canteen policies in each state was from 5% and up to 62%. In the majority of states, 35% of schools achieved compliance [15,16]. 

In the present study, elementary schools were more likely to comply with criteria on minimum frequency of serving high-fat meat as the main protein component and maximum frequency of serving non-wholegrain products when compared with upper secondary schools. In line with this, Girano et al. found that compliance was higher in primary schools compared with secondary schools [18]. Young people/adolescents are at a transition stage between a childhood eating pattern, mainly controlled by parents, and an independent adulthood eating pattern. The changes in food consumption that occur during this transition often lead to a decrease in the overall diet quality. Maintaining a healthy food environment during this transition, especially at schools, may help young people to keep or adopt healthy dietary behaviours [57]. 

There are few studies on compliance with food service guidelines in workplace canteens. A study by Miller et al. found that 25% out of 278 Australian health facility managers providing food and drinks to staff, visitors and the general public reported full implementation of A Better Choice, that classifies foods and drinks into three colour-coded categories: “Green” (best choices), “Amber” (choose carefully) and “Red” (limit), in all food supply areas in which it applied [58]. 

Similarly, the present study showed that 13% complied to all five criteria leaving room for improvement in the workplace canteens with regard to compliance with the food service guidelines. Nevertheless, workplace canteens were found to be more likely to comply with the criteria of minimum serving of fish and minimum fruit and vegetable content compared with both elementary school and upper secondary school canteens. This might partly be explained by a longer tradition in workplaces having a weekly day with fish, combined with the more challenging task of promoting intake of fish among children in schools in Denmark [59]. Results from the present study on compliance with fruit and vegetable content in hot meals among workplaces are comparable with the study by Thorsen et al. with regard to the evaluation of the nutritional quality of hot meals offered at 553 workplace canteens. A total of 58% of the canteens reported to serve hot meals according to the Danish “Plate model” illustrating a recommended meal composition with 40% fruit and vegetables [51]. In addition, the ‘6-a-day’ campaign, since the year 2000, regarding increasing the fruit and vegetable consumption in Denmark, targeted workplace canteens [60]. Thorsen et al. also suggested a positive relationship between corporate financial support and the availability of healthy meal options [51]. Many workplaces may be willing to support in-house canteens in order to promote workers’ good health and to enhance employees’ productivity and the corporate image [48]. Serving system was found to be associated with compliance with regard to frequency of serving non-wholegrain. Buffet serving as a serving system is widely used at workplaces in Denmark. Buffet serving was in a cross-sectional study among 15 workplace canteens also found to be associated with a significantly higher fruit and vegetable consumption compared with an a la carte system [50].

The present study shows that canteens using a high degree of organic food procurement at elementary schools, upper secondary school and workplaces were more likely to serve healthy hot meals according to the food service guidelines with regard to fruit and vegetables, high-fat meat as main protein component and all five criteria combined. These findings are comparable with a study from 2005 that showed a strong correlation between caterers’ use of organic products, and the nutritional quality of the menu options offered [37]. On the other hand, the present study showed that higher odds of compliance were not seen with regard to the criteria on minimum frequency of serving fish, maximum frequency of serving high-fat dairy/meat products nor maximum frequency of serving non-wholegrain indicating that organic food may be linked to some healthier dietary patterns in favour of plant-based foods, but not all. 

In the present study, the vast majority of canteens reported to have a strong or very strong focus on food waste reduction activities. This is in line with results found in a newly published study of Swedish municipal food service organizations [61]. This possibly reflects that food waste reduction has attracted increasing attention in recent years due to both sustainability and economic incentives [61]. Other former studies have shown different results [62]. It is worth noting that canteens using a high degree of organic food procurement in the present study were significantly more likely to report that they had a very high focus on reducing food waste compared with canteens with a lower use of organic food procurement. Sørensen et al. suggest that food-waste management is necessary to optimize the food production in the kitchens and save resources that may cover the premium prices of organic food [38]. This possibly indicates that canteens with higher organic procurement are first movers on more comprehensive waste reduction. In the present study, canteens using organic procurement were found to have more priorities in optimizing, especially in the use of raw foods compared with those not using organic food procurement to the same extent. Steen et al. found in a study among 177 Swedish school and pre-school catering units that the main factors influencing serving waste and total waste per portion were type of kitchen (production or satellite units) and rate of overproduction, while plate waste was mainly influenced by children’s age and factors indicating a stressful dining environment [45]. Betz et al. points out on the basis of a study of two Swiss companies in the education and business sectors that serving losses, which was the main group of losses and almost completely avoidable, could be minimized by adapting portion size to the customers’ requirements and preferred portion sizes and using smaller serving bowls [53]. A study at an Italian school canteen showed that food waste could be reduced through meal planning and leftover re-use schemes [63]. In line with this, food waste was in the present study mainly reported to be reduced by the canteens by reusing of excess production/leftovers (84%) and by optimizing the use of raw products (i.e., using the entire product, 47%), but also by adapting portion size (34%) and using smaller serving bowls (27%). The EAT-Lancet commission suggests in order to increase sustainability of food consumption that food service operators should minimize food waste through careful planning and portioning and be proactive by using the entire product at every chance [2].

When developing food service guidelines, it is important to consider the environmental impact. The Danish food service guidelines were originally pilot tested in seven canteens, including canteens using organic foods to make sure that fulfilling the guidelines was not hampered by the use of organic foods [64]. Moreover, to prevent that adoption of guidelines would lead to an increase of food waste, the guidelines allow food leftovers to be offered, for example, on the buffet the following day without affecting the evaluation of fulfilling the food service guidelines. 

The study has both strengths and limitations to consider. A strength of the present survey is that the data collection was conducted nationwide, which increases generalizability of our study findings. In addition, it allows for a comparison of compliance with hot meal guidelines in different settings, as the criteria for this meal category is the same across settings. This study also presents some limitations. First, the relatively low participation rate (34%) might lead to, e.g., an overestimation of compliance as a result of participants being more health conscious than the rest of the canteens. The willingness to participate in surveys might be limited due to canteen managers being very busy, and a similar low response rate, 29%, was reported in a former canteen survey from Denmark [51]. Furthermore, our results are limited by the self-reported nature of all the information obtained, which are susceptible to recall and social desirability bias. Importantly, the subjective question regarding focus on food waste cannot provide information about actual amounts of food waste but rather on awareness and attitudes towards food waste reduction. Also, it is acknowledged that implementing food service guidelines is a process that may take time. Food offerings need to be adapted to customers’ preferences, and selections offered from retailers can be inadequate [65]. Since the food service guidelines have been established only quite recently in Denmark, it is possible that compliance will increase over time. Future research could include in-depth interviews with the canteen managers from different settings to gain a better understanding of challenges faced by them in complying with food service guidelines.

## 5. Conclusions

This study is the first, to our knowledge, to determine compliance with food service guidelines and to focus on food waste across both the school and workplace settings. The majority of the canteens did not report fully complying with the guidelines on hot meals. Overall, compliance with the guideline was more likely among canteens with a high degree of organic food procurement. Moreover, compliance with the individual hot meal criteria varied widely across settings, with elementary schools being more likely to comply with the criteria on, e.g., maximum serving of non-wholegrain products and high-fat meat, whereas workplaces were more likely to comply with the criteria on minimum fruit and vegetable content of the meals and serving of fish. The study moreover showed that a vast majority of the canteens have a strong self-evaluated focus in food waste reducing activities. Canteens with a high degree of organic food procurement and a written meal policy were more likely to have a very high degree of focus in food waste reduction than other canteens. These findings highlight the need to address differences in canteen characteristics when planning implementation support for both food service guidelines and initiatives on food waste reduction to contribute to the achievement of the UN’s Sustainable Development Goals.

## Figures and Tables

**Table 1:** Danish food service guidelines for hot meals served at lunch in elementary schools, upper secondary schools and workplaces [24,25].

Criteria	Description
Minimum fruit and vegetable content in the meals	Fruit and vegetables account for at least ⅓ of the whole hot meal. Once a week ^1^, fruit and vegetables can account for less than ⅓ of the hot meals if a buffet of salad is offered as part of the lunch options.
Minimum frequency of serving fish and fish products	Fish must be served at least once a week ^1^
Maximum frequency of serving high-fat meat (main protein component) ^2^	Meat products with a high content of fat (>10% fat) can be served in up to 1 of 5 hot meals as main protein component of the meal.
Maximum frequency of serving non-wholegrain products	Grain products with little or no wholegrain (non-wholegrain products) can be served in up to 1 of 5 hot meals.
Maximum frequency of using high-fat dairy/meat products in the meals (limited quantities) ^2^	High-fat meat and dairy based products (meat > 10% fat, cheese > 17% fat, milk > 5% fat) in limited quantities can be served in up to 2 of 5 hot meals.

^1^ As most canteens serve hot meals 5 days a week this most often equals 1 in 5 days; ^2^ There are two criteria for serving high-fat meat. One for meat as a main protein component and one for meat used in limited quantities, e.g., bacon.

**Table 2:** Characteristics of the participating canteens.

Characteristics	Elementary Schools (*n* = 170)		Upper Secondary Schools (*n* = 94)		Workplaces (*n* = 416)		All (*n* = 680)	
	n	%	n	%	N	%	n	%
Number of daily lunch meals (<100)	91	54	39	41	198	48	328	48
Outsourced to external contractors (yes)	8	5	22	23	124	30	154	23
Written canteen meal policy (yes)	97	57	45	48	161	39	303	45
Serving system (buffet style)	46	28	64	69	374	91	484	71
Organic food procurement (>50%)	38	22	8	9	46	11	92	14

**Table 3:** Compliance with the guidelines for hot meals.

Criteria	Elementary Schools (*n* = 120)		Upper Secondary Schools (*n* = 86)		Workplaces (*n* = 383)		All (*n* = 589)	
	n	%	n	%	n	%	n	%
Minimum fruit and vegetable content	45	38	27	31	171	45	243	41
Minimum frequency of serving fish	74	62	43	50	315	82	432	73
Maximum frequency of serving high- fat meat as main protein component	106	88	70	81	266	69	442	75
Maximum frequency of serving non-whole-grain products	96	80	57	66	250	65	403	68
Maximum frequency of using high-fat dairy/meat products in the meals	74	62	46	53	172	45	292	50
All (5 criteria combined)	23	19	9	10	49	13	81	14

**Table 4:** Variables significantly (*P* ≤ 0.05) associated with compliance with guidelines for hot meals ^1^.

Criteria	Significant Variables	OR (95% CI)	*p* Value ^2^
Minimum fruit and vegetable content (*n* = 514)	Setting overall		0.004
	Workplace v. elementary school	1.84 (1.16,2.90)	0.009
	Workplace v. upper secondary school	1.98 (1.17,3.34)	0.011
	Elementary school v. upper secondary school	1.08 (0.58,2.01)	0.818
	Organic food procurement >50% v. <50%	1.88 (1.14,3.11)	0.014
Minimum frequency of serving fish as main protein component (*n* = 566)	Setting overall		<0.0001
	Workplace v. elementary school	3.58 (2.19,5.84)	<0.0001
	Workplace v. upper secondary school	5.63 (3.35,9.45)	<0.0001
	Elementary school v. upper secondary school	1.57 (0.87,2.84)	0.133
Maximum frequency of serving high-fat meat as main protein component (*n* = 511)	Setting overall		0.000
	Workplace v. elementary school	0.18 (0.08,0.43)	<0.0001
	Workplace v. upper secondary school	0.50 (0.26,0.97)	0.042
	Elementary school v. upper secondary school	2.77 (1.08,7.11)	0.035
	Organic food procurement >50% v. <50%	2.78 (1.27,6.09)	0.011
	Meal policy v. no meal policy	0.62 (0.40,0.96)	0.033
	Buffet v. no buffet style	2.06 (1.08,3.92)	0.029
Maximum frequency of serving non-wholegrain products (*n* = 562)	Setting overall		0.005
	Workplace v. elementary school	0.34 (0.18,0.66)	0.001
	Workplace v. upper secondary school	0.89 (0.52,1.53)	0.681
	Elementary school v. upper secondary school	2.62 (1.28,5.37)	0.009
	Buffet v. no buffet style	2.47 (1.44,4.24)	0.001
	Outsourced v. not outsourced	0.50 (0.33,0.75)	0.001
Maximum frequency of using high-fat dairy/meat products in the meals (*n* = 535)	Setting overall		0.004
	Workplace v. elementary school	0.47 (0.30,0.74)	0.001
	Workplace v. upper secondary school	0.73 (0.45,1.19)	0.210
	Elementary school v. upper secondary school	1.56 (0.86,2.84)	0.144
All (5 guidelines combined) (*n* = 542)	Organic food procurement >50% v. <50%	2.00 (1.13,3.53)	0.017

OR, odds ratio; CI, confidence interval; ^1^ Canteens serving hot meals at lunch at least 3 times a week were included in the analysis (*n* = 589); ^2^ Tested using multiple logistic regression. Variables included: Setting, number of daily lunch meals, outsourced to external contractors, written canteen meal policy, serving system, organic food procurement.

**Table 5:** Canteens’ focus on food waste reduction.

Response Categories	Elementary Schools (*n* = 170)		Upper Secondary School (*n* = 94)		Workplaces (*n* = 416)		All (*n* = 680)	
	n	%	n	%	n	%	n	%
To what extent does the canteen focus on food waste reduction?								
To a very low degree	6	4	3	3	11	3	20	3
To a low degree	2	1	0	0	4	1	6	1
Somewhat	13	8	7	7	35	8	55	8
To a high degree	35	21	28	30	117	28	180	26
To a very high degree	106	62	55	59	242	58	403	59
Do not know	5	3	0	0	0	0	5	1
Unanswered	3	2	1	1	7	2	11	2

**Table 6:** Variables significantly (*p* ≤ 0.05) associated with a very high focus on food waste reduction.

Focus Point	Significant Variables	OR (95% CI)	*p* Value ^1^
Food waste (*n* = 584)	Organic food procurement >50% v. <50%	1.91 (1.12,3.25)	0.017
	Meal policy v. no meal policy	1.84 (1.31,2.59)	0.000

OR, odds ratio; CI, confidence interval; ^1^ Tested using multiple logistic regression. Variables included: Setting, number of daily lunch meals, outsourced to external contractors, written canteen meal policy, serving system, organic food procurement.

## Supplementary Materials

The following are available online at https://www.mdpi.com/1660-4601/16/7/1115/s1, S1–S3: Survey questionnaires for elementary schools, upper secondary schools and workplaces. Table S1: Distribution on the Danish regions by invited and participating schools and workplaces in the survey.
