# Supplementary material for: Characteristics of Canteens at Elementary Schools, Upper Secondary Schools and Workplaces that Comply with Food Service Guidelines and Have a Greater Focus on Food Waste
Source: Int J Environ Res Public Health. 2019 Mar 28;16(7):1115. doi: 10.3390/ijerph16071115 (PMC6479865; doi:10.3390/ijerph16071115)
Supplement: Supplementary file 1 [file ijerph-16-01115-s001.pdf]

Supplementary Materials
S1: Survey questionnaire for elementary schools [in danish]
(questions relevant for this article are shown, questions marked with * are compulsory)

Om skolen

1 []

Hvilken type skole er det? *

Vælg ét af følgende mulige svar:

- Folkeskole
- Privatskole/friskole
- Ved ikke
- Andet

2 []Hvilken kommune ligger skolen i? *

Vælg ét af følgende mulige svar:

- Albertslund Kommune
- ...
- Ærø Kommune

3 []Hvor mange elever er der på skolen? *

Vælg ét af følgende mulige svar:

- under 50
- ca. 50-100
- ca. 100-200
- ca. 200-500
- ca. 500-1000
- over 1000
- Ved ikke

Overordnet om måltider og antal kunder

4 []

Hvor hyppigt tilbydes mad eller måltider på følgende tidspunkter?
(Sæt ét kryds for hvert tidspunkt på dagen)

Vælg ét svar for hvert udsagn:

	Hver dag (5 til 7 gange om ugen)	3-4 gange om ugen	1-2 gange om ugen	Hver anden uge	Aldrig/ sjældent	Ved ikke
Morgenmad						
Formiddag						
Frokost						
Eftermiddag						
Aftensmad						
Natmad						

5 []

Hvor mange elever serveres der typisk *frokost* til? *

Vælg ét af følgende mulige svar:

- under 25
- ca. 25-50
- ca. 50-100
- ca. 100-200
- ca. 200-500
- ca. 500-1000
- over 1000
- Ved ikke

6 []Hvordan tilbydes *frokosten* fortrinsvis? *

Vælg én eller flere:

- Buffet/tag selv
  (frit valg mellem fx varm ret, salat- og/eller pålægsbufffet)
- Salg af enkelte tilbud hver for sig
  (fx seperat pris for varm ret, salatbuffet mm)
- Portionsanrettet eller samlet menu
  (fx tallerken med bolle, pålæg og snackgrønt)
- Bordservering
  (fx fade der sættes på spisebordet)
- Ved ikke
- Andet: ____________

Frokost, inklusiv formiddag og eftermiddag

7 []

Hvilke af følgende madtilbud er der typisk til frokost, inklusiv formiddag og eftermiddag?
(Bagefter spørger vi mere detaljeret til de forskellige tilbud og til andre mad-, snack og drikketilbud)
*

Vælg én eller flere:

- ☐ Frokostretter, fx varme retter
  ("dagens ret", vegetarretter eller andre retter, der kan udgøre et helt måltid)
- ☐ Småretter
  (fx pølsehorn, pizzasnegl, toast)
- ☐ Sandwich
- ☐ Wraps o.l.
- ☐ Smurt smørrebrød
- ☐ Pålægs- og brødbuffet
  (dvs. forskellige slags pålæg og brød som ta' selv)
- ☐ Brød og/eller pålæg i "løssalg"
  (fx boller og ost solgt enkeltvis)
- ☐ Salatbuffet
- ☐ Ingen af disse
- ☐ Ved ikke
- ☐ Andet: ____________

Frokostretter, fx varme retter

8 []

Hvor mange dage om ugen tilbydes frokostretter, fx varme retter?
("Dagens ret", vegetarretter eller andre retter, der kan udgøre et helt måltid) *

Vælg ét af følgende mulige svar:

- Hver dag (5-7 gange om ugen)
- 3-4 gange om ugen
- 1-2 gange om ugen
- Hver anden uge
- Aldrig/sjældent
- Ved ikke

9 []

Hvor mange forskellige slags frokostretter tilbydes typisk per dag? *

Vælg ét af følgende mulige svar:

- 1 frokostret dagligt
- 2 slags frokostretter dagligt
- 3 slags frokostretter dagligt
- 4 slags frokostretter dagligt
- 5 slags frokostretter dagligt eller flere
- Ved ikke

Eksempel på besvarelse: Hvis en kantine dagligt tilbyder dagens ret og en suppe, så er svaret "2 slags frokostretter dagligt"

10 []

Hvilke typer frokostretter blev tilbudt den seneste uge?
(Sæt ét kryds for hver dag) *

Vælg ét svar for hvert udsagn:

	Kød, stivelse og grønt hver for sig (fx bøf med kartofler og løg)	Sammen-kogt/samlet ret (fx gryderet, pizza, lasagne)	Suppe (fx hønsekøds- og tomatsuppe)	Grød (fx risengrød, byggrød)	Salat som helt måltid (fx pastasalat med kylling)	Ingen af disse rettyper	Ikke relevant/ Ved ikke
Mandag							
Tirsdag							
Onsdag							
Torsdag							
Fredag							

11 []Hvad var rettens hoved proteinkilde, dvs. kød, fisk, æg, bælgfrugter o.l.?
(Sæt ét kryds for hver dag) *

Vælg ét svar for hvert udsagn:

	Fjerkræ (fx kylling, kalkun)	Rødt kød* *over* 10% fedt (fx svinekam, tykkam, lammekølle)	Rødt kød* *højst* 10% fedt (fx mørbrad, hakket kød 4-10%)	Indmad (fx lever, hjerte)	Fisk og skaldyr	Æg	Bælg-frugter (fx bønner, linser)	Ingen af disse produkter	Ikke relevant/ Ved ikke
Mandag									
Tirsdag									
Onsdag									
Torsdag									
Fredag									

* Rødt kød er kød fra firbenede dyr, fx svin, okse, lam, vildt

12 []Hvad var rettens hoved stivelseskilde, dvs. kornprodukter og kartofler?
(Sæt ét kryds for hver dag) *

Vælg ét svar for hvert udsagn:

	Kartofler	Hvide ris, couscous o.l.	Fuldkorns ris, ker-ner o.l.*	Pasta o.l. uden fuldkorn	Fuldkorns-pasta o.l. *	Ikke-fuldkorns brød o.l.	Fuldkorns-brød o.l.*	Ingen af disse produkter	Ikke relevant/ Ved ikke

	Kartofler	Hvide ris, couscous o.l.	Fuldkorns ris, kerner o.l.*	Pasta o.l. uden fuldkorn	Fuldkorns-pasta o.l. *	Ikke-fuldkorns brød o.l.	Fuldkorns-brød o.l.*	Ingen af disse produkter	Ikke relevant/ Ved ikke
Mandag									
Tirsdag									
Onsdag									
Torsdag									
Fredag									

* Dvs. lever op til Nøglehulsmærket eller Fuldkornsmærket. For hjemmebagt brød svarer det til, at mindst en tredjedel af melet er fuldkorn.

13 []

Hvor meget fyldte grønsager og frugt i forhold til den samlede frokostret ved servering?

(Medregn al frugt og grønt i retten inklusiv bælgfrugter men *ikke* kartofler og *ikke* salatbuffet)

(Sæt ét kryds for hver dag) *

Vælg ét svar for hvert udsagn:

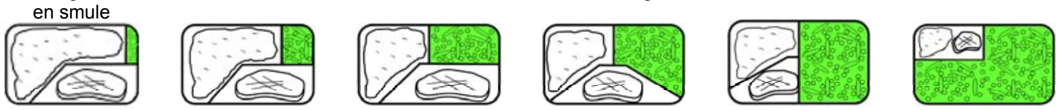

	Ikke noget eller en smule	En mindre del	En god del	En væsentlig del	Over halvdelen	Størstedelen	Ikke relevant/ Ved ikke
Mandag							
Tirsdag							
Onsdag							
Torsdag							
Fredag							

14 []

Hvilke af følgende federe produkter indeholdt retten?

(Sæt ét eller flere krydser for hver dag) *

	Hårde fedtstoffer* (fx smør, hård margarine, kokosmælk)	Mejeri-produkter *over* 5% fedt (fx creme fraiche, fløde)	Ost *over* 17% fedt (fx fast ost 45+, klassisk salatost)	Federe kød som smagsgiver (fx bacon, chorizo)	Planteolier o.l.* (fx olie, blød margarine, pesto)	Ingen af disse produkter	Ikke relevant/ Ved ikke
Mandag							
Tirsdag							
Onsdag							
Torsdag							
Fredag							

* Fedtstoffer til stegning tæller kun med, hvis de indgår i retten, dvs. hvis stegefedtet ikke smides ud

15 []

I forhold til den beskrevne ret, hvordan adskiller den anden frokostret sig? *

Vælg én eller flere:

- Vegetarisk
- Lidt mindre portion (evt. lavere pris)
- Tilpasset særlige behov (allergi, særlige ønsker mm)
- Veksler afhængig af tilbud/eventuelle rester
- Salat serveret som helt måltid
- Adskiller sig ikke
- Ved ikke
- Andet: ____________

16 []Indeholdt den anden frokostret fisk eller skaldyr én eller flere af dagene (mandag til fredag)? *

Vælg ét af følgende mulige svar:

- Ja
- Nej
- Ved ikke

Rammer og praksis

17 []

Hvor mange ansatte er der i køkkenet omregnet til fuldtidsansatte? *

Vælg ét af følgende mulige svar:

- 0
- ca. 0,5
- ca. 1
- ca. 2
- ca. 3
- ca. 4
- ca. 5
- ca. 6
- ca. 7
- ca. 8
- ca. 9
- ca. 10
- ca. 11-15
- ca. 16-20
- ca. 21-25
- over 25
- Ved ikke

18 []

Hvilken uddannelse har køkkenets medarbejdere?

Vælg én eller flere:

- Ernærings- eller køkkenassistent
- Ernæringsteknolog
- Køkkenleder
- Økonoma
- Cater
- Kok
- Bager/konditor
- Slagter
- Professionsbachelor i ernæring og sundhed
- Ufaglært
- Anden kostfaglig uddannelse
- Ved ikke
- Andet: ____

19 []Er madordningen drevet af en ekstern kantineoperatør? *

Vælg ét af følgende mulige svar:

- Ja
- Nej
- Ved ikke
- Andet ____

20 []Yder skolen tilskud til løn og/eller råvarer? *

Vælg ét af følgende mulige svar:

- Ja, til råvarer
- Ja, til løn
- Ja, til både råvarer og løn
- Nej

Ved ikke

Andet

21 []

Hvor stor en andel af køkkenets produkter er økologiske?
(Angiv procent ud fra bedste skøn eller målt) *

Vælg ét af følgende mulige svar:

- ca. 0-10%
- ca. 10-20%
- ca. 20-30%
- ca. 30-40%
- ca. 40-50%
- ca. 50-60%
- ca. 60-70%
- ca. 70-80%
- ca. 80-90%
- ca. 90-100%
- Ved ikke

22 []Har køkkenet Det Økologiske Spisemærke? *

Vælg ét af følgende mulige svar:

- Ja, Det Økologiske Spisemærke i Bronze
- Ja, Det Økologiske Spisemærke i Sølv
- Ja, Det Økologiske Spisemærke i Guld
- Nej
- Ved ikke

23 []Har skolen en nedskreven mad- og måltidspolitik? *

Vælg ét af følgende mulige svar:

- Ja
- Nej
- Ved ikke
- Andet

24 []Hvem har udarbejdet den? *

Vælg én eller flere:

- Køkkenet
- Kommunen
- Skolen
- Kantineoperatøren
- Forældrene
- Ved ikke
- Andet:

25 []I hvor høj grad har køkkenet fokus på at:

Vælg ét svar for hvert udsagn:

	I meget lav grad	I lav grad	I nogen grad	I høj grad	I meget høj grad	Ved ikke
Begrænse madspild						
Begrænse salt i maden						
Begrænse søde sager						

26 []Hvad gør I for at begrænse madspild?

Vælg én eller flere:

- Udnytter alle rester
- Sætter mindre mad frem ad gangen

- [ ] Bruger hele råvaren op
- [ ] Tilpasser portionsstørrelserne
- [ ] Udportionerer i mindre skåle/portioner
- [ ] Sælger "rester" til ansatte, fx til at tage med hjem
- [ ] Andet: ____________________

Kort om dig selv

27 []

Hvad er dit køn? *

Vælg ét af følgende mulige svar:

- Kvinde
- Mand

28 []

**Hvad er din funktion på skolen?
****

Vælg ét af følgende mulige svar:

- Køkkenchef eller bestyrer af madordningen
- Medarbejder i køkkenet
- Leder/afdelingschef o.l. på skolen
- Andet

29 []Hvis du har øvrige kommentarer til indholdet eller udfyldelsen af spørgeskemaet, kan de skrives her:

Skriv dit svar her:

30-11-2017 – 13:07

Indsend spørgeskema
Tak for din deltagelse i undersøgelsen.

Om skolen

1 []

Hvilken type skole er det? *

Vælg ét af følgende mulige svar:

- Gymnasial uddannelse (STX, HF, HTX, HHX mv.)
- Erhvervsuddannelse (EUD mv.)
- Kombination af gymnasial uddannelse og erhvervsuddannelse
- Produktionsskole
- Ved ikke
- Andet

2 []Hvilken kommune ligger skolen i? *

Vælg ét af følgende mulige svar:

- Albertslund Kommune
- ...
- Ærø Kommune

3 []Hvor mange elever er der på skolen? *

Vælg ét af følgende mulige svar:

- under 50
- ca. 50-100
- ca. 100-200
- ca. 200-500
- ca. 500-1000
- over 1000
- Ved ikke

Overordnet om måltider og antal kunder

4 []

Hvor hyppigt tilbydes mad eller måltider på følgende tidspunkter?
(Sæt ét kryds for hvert tidspunkt på dagen)

Vælg ét svar for hvert udsagn:

	Hver dag (5 til 7 gange om ugen)	3-4 gange om ugen	1-2 gange om ugen	Hver anden uge	Aldrig/ sjældent	Ved ikke
Morgen						
Formiddag						
Frokost						
Eftermiddag						
Aften						
Nat						

5 []

Hvor mange elever serveres der typisk *frokost* til? *

Vælg ét af følgende mulige svar:

- under 25
- ca. 25-50
- ca. 50-100
- ca. 100-200
- ca. 200-500
- ca. 500-1000
- over 1000
- Ved ikke

6 []Hvordan tilbydes *frokosten* fortrinsvis? *

Vælg én eller flere:

- Buffet/tag selv
  (frit valg mellem fx varm ret, salat- og/eller pålægsbufffet)
- Salg af enkelte tilbud hver for sig
  (fx seperat pris for varm ret, salatbuffet mm)
- Portionsanrettet eller samlet menu
  (fx tallerken med bolle, pålæg og snackgrønt)
- Bordservering
  (fx fade der sættes på spisebordet)
- Ved ikke
- Andet: ____________

Frokost, inklusiv formiddag og eftermiddag

7 []

Hvilke af følgende madtilbud er der typisk til frokost, inklusiv formiddag og eftermiddag?
(Bagefter spørger vi mere detaljeret til de forskellige tilbud og til andre mad-, snack og drikketilbud)
*

Vælg én eller flere:

- ☐ Frokostretter, fx varme retter
  ("dagens ret", vegetarretter eller andre retter, der kan udgøre et helt måltid)
- ☐ Småretter
  (fx pølsehorn, pizzasnegl, toast)
- ☐ Sandwich
- ☐ Wraps o.l.
- ☐ Smurt smørrebrød
- ☐ Pålægs- og brødbuffet
  (dvs. forskellige slags pålæg og brød som ta' selv)
- ☐ Brød og/eller pålæg i "løssalg"
  (fx boller og ost solgt enkeltvis)
- ☐ Salatbuffet
- ☐ Ingen af disse
- ☐ Ved ikke
- ☐ Andet: ____________

Frokostretter, fx varme retter

8 []

Hvor mange dage om ugen tilbydes frokostretter, fx varme retter?
("Dagens ret", vegetarretter eller andre retter, der kan udgøre et helt måltid) *

Vælg ét af følgende mulige svar:

- Hver dag (5-7 gange om ugen)
- 3-4 gange om ugen
- 1-2 gange om ugen
- Hver anden uge
- Aldrig/sjældent
- Ved ikke

9 []

Hvor mange forskellige slags frokostretter tilbydes typisk per dag? *

Vælg ét af følgende mulige svar:

- 1 frokostret dagligt
- 2 slags frokostretter dagligt
- 3 slags frokostretter dagligt
- 4 slags frokostretter dagligt
- 5 slags frokostretter dagligt eller flere
- Ved ikke

Eksempel på besvarelse: Hvis en kantine dagligt tilbyder dagens ret og en suppe, så er svaret "2 slags frokostretter dagligt"

10 []Hvilke typer frokostretter blev tilbudt den seneste uge?
(Sæt ét kryds for hver dag) *

Vælg ét svar for hvert udsagn:

	Kød, stivelse og grønt hver for sig (fx bøf med kartofler og løg)	Sammenkogt/samlet ret (fx gryderet, pizza, lasagne)	Suppe (fx hønsekøds- og tomatsuppe)	Grød (fx risengrød, byggrød)	Salat som helt måltid (fx pastasalat med kylling)	Ingen af disse rettyper	Ikke relevant/ Ved ikke
Mandag							
Tirsdag							
Onsdag							
Torsdag							
Fredag							

11 []Hvad var rettens hoved proteinkilde, dvs. kød, fisk, æg, bælgfrugter o.l.?
(Sæt ét kryds for hver dag) *

Vælg ét svar for hvert udsagn:

	Fjerkræ (fx kylling, kalkun)	Rødt kød* *over* 10% fedt (fx svinekam, tykkam, lammekølle)	Rødt kød* *højst* 10% fedt (fx mørbrad, hakket kød 4-10%)	Indmad (fx lever, hjerte)	Fisk og skaldyr	Æg	Bælgfrugter (fx bønner, linser)	Ingen af disse produkter	Ikke relevant/ Ved ikke
Mandag									
Tirsdag									
Onsdag									
Torsdag									
Fredag									

* Rødt kød er kød fra firbenede dyr, fx svin, okse, lam, vildt

12 []Hvad var rettens hoved stivelseskilde, dvs. kornprodukter og kartofler?
(Sæt ét kryds for hver dag) *

Vælg ét svar for hvert udsagn:

	Kartofler	Hvide ris, couscous o.l.	Fuldkorns ris, kerner o.l.*	Pasta o.l. uden fuldkorn	Fuldkornspasta o.l. *	Ikke-fuldkorns brød o.l.	Fuldkornsbrød o.l.*	Ingen af disse produkter	Ikke relevant/ Ved ikke
Mandag									
Tirsdag									

	Kartofler	Hvide ris, couscous o.l.	Fuldkorns ris, ker-ner o.l.*	Pasta o.l. uden fuldkorn	Fuldkorns-pasta o.l. *	Ikke-fuldkorns brød o.l.	Fuldkorns-brød o.l.*	Ingen af disse produkter	Ikke relevant/ Ved ikke
Onsdag									
Torsdag									
Fredag									

* Dvs. lever op til Nøglehulsmærket eller Fuldkornsmærket. For hjemmebagt brød svarer det til, at mindst en tredjedel af melet er fuldkorn.

13 []

Hvor meget fyldte grønsager og frugt i forhold til den samlede frokostret ved servering?
(Medregn al frugt og grønt i retten inklusiv bælgfrugter men *ikke* kartofler og *ikke* salatbuffet)

(Sæt ét kryds for hver dag) *

Vælg ét svar for hvert udsagn:

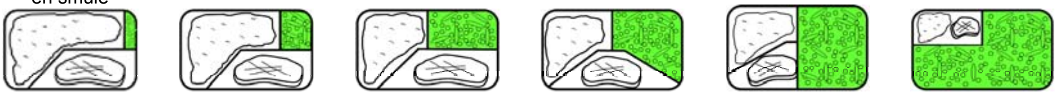

	Ikke noget eller en smule	En mindre del	En god del	En væsentlig del	Over halvdelen	Størstedelen	Ikke relevant/ Ved ikke
Mandag							
Tirsdag							
Onsdag							
Torsdag							
Fredag							

14 []

Hvilke af følgende federe produkter indeholdt retten?
(Sæt ét eller flere krydser for hver dag) *

	Hårde fedtstoffer* (fx smør, hård margarine, kokosmælk)	Mejeri-produkter *over* 5% fedt (fx creme fraiche, fløde)	Ost *over* 17% fedt (fx fast ost 45+, klassisk salatost)	Federe kød som smagsgiver (fx bacon, chorizo)	Planteolier o.l.* (fx olie, blød margarine, pesto)	Ingen af disse produkter	Ikke relevant/ Ved ikke
Mandag							
Tirsdag							
Onsdag							
Torsdag							
Fredag							

* Fedtstoffer til stegning tæller kun med, hvis de indgår i retten, dvs. hvis stegefedtet ikke smides ud

15 []

I forhold til den beskrevne ret, hvordan adskiller den anden frokostret sig? *

Vælg én eller flere:

- Vegetarisk
- Lidt mindre portion (evt. lavere pris)
- Tilpasset særlige behov (allergi, særlige ønsker mm)
- Veksler afhængig af tilbud/eventuelle rester
- Salat serveret som helt måltid
- Adskiller sig ikke
- Ved ikke
- Andet: ____________

16 []Indeholdt den anden frokostret fisk eller skaldyr én eller flere af dagene (mandag til fredag)? *

Vælg ét af følgende mulige svar:

- Ja
- Nej
- Ved ikke

Rammer og praksis

17 []

Hvor mange ansatte er der i køkkenet omregnet til fuldtidsansatte? *

Vælg ét af følgende mulige svar:

- 0
- ca. 0,5
- ca. 1
- ca. 2
- ca. 3
- ca. 4
- ca. 5
- ca. 6
- ca. 7
- ca. 8
- ca. 9
- ca. 10
- ca. 11-15
- ca. 16-20
- ca. 21-25
- over 25
- Ved ikke

18 []

Hvilken uddannelse har køkkenets medarbejdere?

Vælg én eller flere:

- Ernærings- eller køkkenassistent
- Ernæringsteknolog
- Køkkenleder
- Økonoma
- Cater
- Kok
- Bager/konditor
- Slagter
- Professionsbachelor i ernæring og sundhed
- Ufaglært
- Anden kostfaglig uddannelse
- Ved ikke
- Andet: ____

19 []Er kantinen drevet af en ekstern kantineoperatør? *

Vælg ét af følgende mulige svar:

- Ja
- Nej
- Ved ikke
- Andet ____

20 []Yder skolen tilskud til løn og/eller råvarer? *

Vælg ét af følgende mulige svar:

- Ja, til råvarer
- Ja, til lønninger
- Ja, til både råvarer og løn
- Nej

- Ved ikke
- Andet [ ]

21 []

Hvor stor en andel af køkkenets produkter er økologiske?
(Angiv procent ud fra bedste skøn eller målt) *

Vælg ét af følgende mulige svar:

- ca. 0-10%
- ca. 10-20%
- ca. 20-30%
- ca. 30-40%
- ca. 40-50%
- ca. 50-60%
- ca. 60-70%
- ca. 70-80%
- ca. 80-90%
- ca. 90-100%
- Ved ikke

22 []Har køkkenet Det Økologiske Spisemærke? *

Vælg ét af følgende mulige svar:

- Ja, Det Økologiske Spisemærke i Bronze
- Ja, Det Økologiske Spisemærke i Sølv
- Ja, Det Økologiske Spisemærke i Guld
- Nej
- Ved ikke

23 []Har skolen en nedskreven mad- og måltidspolitik? *

Vælg ét af følgende mulige svar:

- Ja
- Nej
- Ved ikke
- Andet [ ]

24 []Hvem har udarbejdet den? *

Vælg én eller flere:

- Kantinen/køkkenet
- Kommunen
- Skolen
- Kantineoperatøren
- Ved ikke
- Andet: [ ]

25 []I hvor høj grad har køkkenet fokus på:

Vælg ét svar for hvert udsagn:

	I meget lav grad	I lav grad	I nogen grad	I høj grad	I meget høj grad	Kender ikke/ Ved ikke
Begrænse madspild						
Begrænse salt i maden						
Begrænse søde sager						

26 []Hvad gør I for at begrænse madspild?

Vælg én eller flere:

- Udnytter alle rester
- Sætter mindre mad frem ad gangen
- Bruger hele råvaren op

- ☐ Tilpasser portionsstørrelserne
- ☐ Udportioner i mindre skåle/portioner
- ☐ Sælger "rester" til ansatte, fx til at tage med hjem
- ☐ Andet: ____________

Kort om dig selv

27 []

Hvad er dit køn? *

Vælg ét af følgende mulige svar:

- Kvinde
- Mand

28 []

Hvad er din funktion på skolen? *

Vælg ét af følgende mulige svar:

- Køkkenchef eller bestyrer af kantinen
- Medarbejder i kantinen
- Leder/afdelingschef o.l. på skolen
- Andet

29 []Hvis du har øvrige kommentarer til indholdet eller udfyldelsen af spørgeskemaet, kan de skrives her:

Skriv dit svar her:

29-11-2017 – 10:09

Indsend spørgeskema
Tak for din deltagelse i undersøgelsen.

Om virksomheden

1 []

Hvilken type virksomhed er det? *

Vælg ét af følgende mulige svar:

- Offentlig arbejdsplads
- Privat virksomhed (Produktion)
- Privat virksomhed (Handel/service)
- Ved ikke
- Andet ________

2 []Hvilken kommune ligger virksomheden i? *

Vælg ét af følgende mulige svar:

- Albertslund Kommune
- ...
- Ærø Kommune

3 []Hvor mange ansatte er der på virksomheden? *

Vælg ét af følgende mulige svar:

- under 50
- ca. 50-100
- ca. 100-200
- ca. 200-500
- ca. 500-1000
- over 1000
- Ved ikke

Overordnet om måltider og antal kunder

4 []

Hvor hyppigt tilbydes mad eller måltider på følgende tidspunkter?
(Sæt ét kryds for hvert tidspunkt på dagen)

Vælg ét svar for hvert udsagn:

	Hver dag (5 til 7 gange om ugen)	3-4 gange om ugen	1-2 gange om ugen	Hver anden uge	Aldrig/ sjældent	Ved ikke
Morgenmad	○	○	○	○	○	○
Formiddag	○	○	○	○	○	○
Frokost	○	○	○	○	○	○
Eftermiddag	○	○	○	○	○	○
Aftensmad	○	○	○	○	○	○
Natmad	○	○	○	○	○	○

5 []

Hvor mange kunder serveres der typisk *frokost* til? *

Vælg ét af følgende mulige svar:

- ○ under 25
- ○ ca. 25-50
- ○ ca. 50-100
- ○ ca. 100-200
- ○ ca. 200-500
- ○ ca. 500-1000
- ○ over 1000
- ○ Ved ikke

6 []Hvordan tilbydes *frokosten* fortrinsvis? *

Vælg én eller flere:

- ☐ Buffet/tag selv
  (frit valg mellem fx varm ret, salat- og/eller pålægsbufffet)
- ☐ Salg af enkelte tilbud hver for sig
  (fx seperat pris for varm ret, salatbuffet mm)
- ☐ Portionsanrettet eller samlet menu
  (fx tallerken med bolle, pålæg og snackgrønt)
- ☐ Bordservering
  (fx fade der sættes på spisebordet)
- ☐ Ved ikke
- ☐ Andet: ____________

Frokost, inklusiv formiddag og eftermiddag

7 []

Hvilke af følgende madtilbud er der typisk til frokost, inklusiv formiddag og eftermiddag?
(Bagefter spørger vi mere detaljeret til de forskellige tilbud og til andre mad-, snack og drikketilbud)
*

Vælg én eller flere:

- ☐ Frokostretter, fx varme retter
  ("dagens ret", vegetarretter eller andre retter, der kan udgøre et helt måltid)
- ☐ Småretter
  (fx pølsehorn, pizzasnegl, toast)
- ☐ Sandwich
- ☐ Wraps o.l.
- ☐ Smurt smørrebrød
- ☐ Pålægs- og brødbuffet
  (dvs. forskellige slags pålæg og brød som ta' selv)
- ☐ Brød og/eller pålæg i "løssalg"
  (fx boller og ost solgt enkeltvis)
- ☐ Salatbuffet
- ☐ Ingen af disse
- ☐ Ved ikke
- ☐ Andet: ____________

Frokostretter, fx varme retter

8 []

Hvor mange dage om ugen tilbydes frokostretter, fx varme retter?
("Dagens ret", vegetarretter eller andre retter, der kan udgøre et helt måltid) *

Vælg ét af følgende mulige svar:

- Hver dag (5-7 gange om ugen)
- 3-4 gange om ugen
- 1-2 gange om ugen
- Hver anden uge
- Aldrig/sjældent
- Ved ikke

9 []

Hvor mange forskellige slags frokostretter tilbydes typisk per dag? *

Vælg ét af følgende mulige svar:

- 1 frokostret dagligt
- 2 slags frokostretter dagligt
- 3 slags frokostretter dagligt
- 4 slags frokostretter dagligt
- 5 slags frokostretter dagligt eller flere
- Ved ikke

Eksempel på besvarelse: Hvis en kantine dagligt tilbyder dagens ret og en suppe, så er svaret "2 slags frokostretter dagligt"

10 []Hvilke typer frokostretter blev tilbudt den seneste uge?
(Sæt ét kryds for hver dag) *

Vælg ét svar for hvert udsagn:

	Kød, stivelse og grønt hver for sig (fx bøf med kartofler og løg)	Sammen-kogt/samlet ret (fx gryderet, pizza, lasagne)	Suppe (fx hønsekøds- og tomatsuppe)	Grød (fx risengrød, byggrød)	Salat som helt måltid (fx pastasalat med kylling)	Ingen af disse rettyper	Ikke relevant/ Ved ikke
Mandag	○	○	○	○	○	○	○
Tirsdag	○	○	○	○	○	○	○
Onsdag	○	○	○	○	○	○	○
Torsdag	○	○	○	○	○	○	○
Fredag	○	○	○	○	○	○	○

11 []Hvad var rettens hoved proteinkilde, dvs. kød, fisk, æg, bælgfrugter o.l.?
(Sæt ét kryds for hver dag) *

Vælg ét svar for hvert udsagn:

	Fjerkræ (fx kylling, kalkun)	Rødt kød* *over* 10% fedt (fx svinekam, tykkam, lammekølle)	Rødt kød* *højst* 10% fedt (fx mørbrad, hakket kød 4-10%)	Indmad (fx lever, hjerte)	Fisk og skaldyr	Æg	Bælg-frugter (fx bønner, linser)	Ingen af disse produkter	Ikke relevant/ Ved ikke
Mandag	○	○	○	○	○	○	○	○	○
Tirsdag	○	○	○	○	○	○	○	○	○
Onsdag	○	○	○	○	○	○	○	○	○
Torsdag	○	○	○	○	○	○	○	○	○
Fredag	○	○	○	○	○	○	○	○	○

* Rødt kød er kød fra firbenede dyr, fx svin, okse, lam, vildt

12 []Hvad var rettens hoved stivelseskilde, dvs. kornprodukter og kartofler?
(Sæt ét kryds for hver dag) *

Vælg ét svar for hvert udsagn:

	Kartofler	Hvide ris, couscous o.l.	Fuldkorns-ris, ker-ner o.l.*	Pasta o.l. uden fuldkorn	Fuldkorns-pasta o.l.*	Ikke-fuldkorns brød o.l.	Fuldkorns-brød o.l.*	Ingen af disse produkter	Ikke relevant/ Ved ikke
Mandag	○	○	○	○	○	○	○	○	○
Tirsdag	○	○	○	○	○	○	○	○	○
Onsdag	○	○	○	○	○	○	○	○	○
Torsdag	○	○	○	○	○	○	○	○	○
Fredag	○	○	○	○	○	○	○	○	○

* Dvs. lever op til Nøglehulsmærket eller Fuldkornsmærket. For hjemmebagt brød svarer det til, at mindst en tredjedel af melet er fuldkorn.

13 []

Hvor meget fyldte grønsager og frugt i forhold til den samlede frokostret ved servering?
(Medregn al frugt og grønt i retten inklusiv bælgfrugter men *ikke* kartofler og *ikke* salatbuffet*)*

(Sæt ét kryds for hver dag) *

Vælg ét svar for hvert udsagn:

	Ikke noget eller en smule 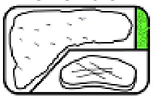	En mindre del 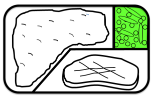	En god del 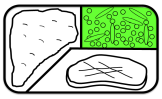	En væsentlig del 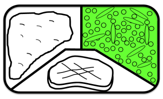	Over halvdelen 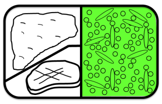	Størstedelen 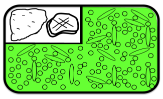	Ikke relevant/ Ved ikke
Mandag	○	○	○	○	○	○	○
Tirsdag	○	○	○	○	○	○	○
Onsdag	○	○	○	○	○	○	○
Torsdag	○	○	○	○	○	○	○
Fredag	○	○	○	○	○	○	○

14 []

Hvilke af følgende federe produkter indeholdt retten?
(Sæt ét eller flere krydser for hver dag) *

	Hårde fedtstoffer* (fx smør, hård margarine, kokosmælk)	Mejeri-produkter *over* 5% fedt (fx creme fraiche, fløde)	Ost *over* 17% fedt (fx fast ost 45+, klassisk salatost)	Federe kød som smagsgiver (fx bacon, chorizo)	Planteolier o.l.* (fx olie, blød margarine, pesto)	Ingen af disse produkter	Ikke relevant/ Ved ikke
Mandag	☐	☐	☐	☐	☐	☐	☐
Tirsdag	☐	☐	☐	☐	☐	☐	☐
Onsdag	☐	☐	☐	☐	☐	☐	☐
Torsdag	☐	☐	☐	☐	☐	☐	☐
Fredag	☐	☐	☐	☐	☐	☐	☐

* Fedtstoffer til stegning tæller kun med, hvis de indgår i retten, dvs. hvis stegefedtet ikke smides ud

15 []

I forhold til den beskrevne ret, hvordan adskiller den anden frokostret sig? *

Vælg én eller flere:

- ☐ Vegetarisk
- ☐ Lidt mindre portion (evt. lavere pris)
- ☐ Tilpasset særlige behov (allergi, særlige ønsker mm)
- ☐ Veksler afhængig af tilbud/eventuelle rester
- ☐ Salat serveret som helt måltid
- ☐ Adskiller sig ikke
- ☐ Ved ikke
- ☐ Andet: ____________

16 []Indeholdt den anden frokostret fisk eller skaldyr én eller flere af dagene (mandag til fredag)? *

Vælg ét af følgende mulige svar:

- ○ Ja
- ○ Nej
- ○ Ved ikke

Rammer og praksis

17 []

Hvor mange ansatte er der i køkkenet omregnet til fuldtidsansatte? *

Vælg ét af følgende mulige svar:

- 0
- ca. 0,5
- ca. 1
- ca. 2
- ca. 3
- ca. 4
- ca. 5
- ca. 6
- ca. 7
- ca. 8
- ca. 9
- ca. 10
- ca. 11-15
- ca. 16-20
- ca. 21-25
- over 25
- Ved ikke

18 []

Hvilken uddannelse har køkkenets medarbejdere?

Vælg én eller flere:

- Ernærings- eller køkkenassistent
- Ernæringsteknolog
- Køkkenleder
- Økonoma
- Cater
- Kok
- Bager/konditor
- Slagter
- Professionsbachelor i ernæring og sundhed
- Ufaglært
- Anden kostfaglig uddannelse
- Ved ikke
- Andet: ____

19 []Er kantinen drevet af en ekstern kantineoperatør? *

Vælg ét af følgende mulige svar:

- Ja
- Nej
- Ved ikke
- Andet ____

20 []Yder virksomheden tilskud til løn og/eller råvarer? *

Vælg ét af følgende mulige svar:

- Ja, til råvarer

- Ja, til løn
- Ja, til både råvarer og løn
- Nej
- Ved ikke
- Andet

21 []

Hvor stor en andel af køkkenets produkter er økologiske?
(Angiv procent ud fra bedste skøn eller målt) *

Vælg ét af følgende mulige svar:

- ca. 0-10%
- ca. 10-20%
- ca. 20-30%
- ca. 30-40%
- ca. 40-50%
- ca. 50-60%
- ca. 60-70%
- ca. 70-80%
- ca. 80-90%
- ca. 90-100%
- Ved ikke

22 []Har køkkenet Det Økologiske Spisemærke? *

Vælg ét af følgende mulige svar:

- Ja, Det Økologiske Spisemærke i Bronze
- Ja, Det Økologiske Spisemærke i Sølv
- Ja, Det Økologiske Spisemærke i Guld
- Nej
- Ved ikke

23 []Har virksomheden en nedskreven mad- og måltidspolitik? *

Vælg ét af følgende mulige svar:

- Ja
- Nej
- Ved ikke
- Andet

24 []Hvem har udarbejdet den? *

Vælg én eller flere:

- Kantinen
- Virksomheden
- Kantineoperatøren
- Ved ikke
- Andet:

25 []I hvor høj grad har køkkenet fokus på at:

Vælg ét svar for hvert udsagn:

	I meget lav grad	I lav grad	I nogen grad	I høj grad	I meget høj grad	Ved ikke
Begrænse madspild	○	○	○	○	○	○
Begrænse salt i maden	○	○	○	○	○	○
Begrænse søde sager	○	○	○	○	○	○

26 []Hvad gør I for at begrænse madspild?

Vælg én eller flere:

- ☐ Udnytter alle rester
- ☐ Sætter mindre mad frem ad gangen
- ☐ Bruger hele råvaren op
- ☐ Tilpasser portionsstørrelserne
- ☐ Udportioner i mindre skåle/portioner
- ☐ Sælger "rester" til ansatte, fx til at tage med hjem
- ☐ Andet: ________

Kort om dig selv

27 []

Hvad er dit køn? *

Vælg ét af følgende mulige svar:

- Kvinde
- Mand

28 []

Hvad er din funktion på virksomheden? *

Vælg ét af følgende mulige svar:

- Køkkenchef eller bestyrer af kantinen
- Medarbejder i kantinen
- Leder/afdelingschef o.l i virksomheden
- Andet

29 []Hvis du har øvrige kommentarer til indholdet eller udfyldelsen af spørgeskemaet, kan de skrives her:

Skriv dit svar her:

10-12-2017 – 15:48

Indsend spørgeskema
Tak for din deltagelse i undersøgelsen.

Supplementary Materials

Table S1. Distribution on the Danish regions by invited and participating schools and workplaces in the survey.

Region	Elementary schools		Upper secondary schools		Workplaces		All	
	Invited (n=367)	Participated (n=170)	Invited (n=208)	Participated (n=94)	Invited (n=1420)	Participated (n=416)	Invited (n=1995)	Participated (n=680)
	%	%	%	%	%	%	%	%
Capital	20	15	31	22	41	34	36	28
Central Denmark	26	31	26	30	23	26	24	28
North Denmark	19	18	6	6	8	9	10	11
Zealand	13	15	16	17	9	11	10	13
Southern Denmark	22	21	21	25	19	20	20	21
